# Supplementary material for: Using Fitbit data to examine factors that affect daily activity levels of college students
Source: PLoS One. 2021 Jan 6;16(1):e0244747. doi: 10.1371/journal.pone.0244747 (PMC7787529; doi:10.1371/journal.pone.0244747)
Supplement: S1 Table — (DOCX) [file pone.0244747.s001.docx]

**S1 Table. Items for Measuring the Big Five Factors in Personality Trait Ratings.**

| Extraversion | 1. I am talkative. |
| --- | --- |
|  | 2. I am reserved. (reversed) |
|  | 3. I am full of energy. |
|  | 4. I generate a lot of enthusiasm. |
|  | 5. I tend to be quiet. (reversed) |
|  | 6. I have an assertive personality. |
|  | 7. I am sometimes shy, inhibited. (reversed) |
|  | 8. I am outgoing, sociable. |
| Agreeableness | 1. I tend to find fault with others. (reversed) |
|  | 2. I am helpful and unselfish with others. |
|  | 3. I start quarrels with others. (reversed) |
|  | 4. I have a forgiving nature. |
|  | 5. I am generally trusting. |
|  | 6. I can be cold and aloof. (revered) |
|  | 7. I am considerate and kind to almost everyone. |
|  | 8. I am sometimes rude to others. (reversed) |
|  | 9. I like to cooperate with others. |
| Conscientiousness | 1. I do a thorough job. |
|  | 2. I can be somewhat careless. (reversed) |
|  | 3. I am a reliable worker. |
|  | 4. I tend to be disorganized. (reversed) |
|  | 5. I tend to be lazy. (reversed) |
|  | 6. I persevere until the task is finished. |
|  | 7. I do things efficiently. |
|  | 8. I make plans and follows through with them. |
|  | 9. I am easily distracted. (reversed) |
| Neuroticism | 1. I am depressed, blue. |
|  | 2. I am relaxed, handles stress well. (reversed) |
|  | 3. I can be tense. |
|  | 4. I worry a lot. |
|  | 5. I am emotionally stable, not easily upset. (reversed) |
|  | 6. I can be moody. |
|  | 7. I remains calm in tense situations. (reversed) |
|  | 8. I get nervous easily. |
| Openness | 1. I am original, comes up with new ideas. |
|  | 2. I am curious about many different things. |
|  | 3. I am ingenious, a deep thinker. |
|  | 4. I have an active imagination. |
|  | 5. I am inventive. |
|  | 6. I value artistic, aesthetic experiences. |
|  | 7. I prefer work that is routine. (reversed) |
|  | 8. I like to reflect, play with ideas. |
|  | 9. I have few artistic interests. (reversed) |
|  | 10. I am sophisticated in art, music, or literature. |
