# Supplementary material for: Using Fitbit data to examine factors that affect daily activity levels of college students
Source: PLoS One. 2021 Jan 6;16(1):e0244747. doi: 10.1371/journal.pone.0244747 (PMC7787529; doi:10.1371/journal.pone.0244747)
Supplement: Attachment — Submitted filename: Response to Reviewers.docx [file pone.0244747.s003.docx]

**Response to Reviewers**

PONE-D-20-21952R1

Using Fitbit Data to Examine Factors that Affect Daily Activity Levels of College Students

PLOS ONE

We would like to thank the reviewers for their very constructive feedback. Incorporating the reviewers’ suggestions for revision has resulted in a greatly improved manuscript. Below, we note the concerns of the reviewers and then explain how we responded to each comment. In all cases, the reviewers’ comments are in italics, while our own comments are in plain text.

**Reviewer 1**

*1. Please include the average and range (max and min) for the number of days of data included in the model for subjects participating in this study. This should be average, min and max number of days for participants after exclusion of days that did not meet the 80% threshold.*

––––––––––––––––––––––––––––––––––––––––––––––––––––

Thanks for the suggestion. After applying the 80% threshold, the number of days of Fitbit data ranges from 1 to 278, with a mean of 148 and standard deviation of 75. We now report these numbers on page 12 in the revised manuscript.

*2.1 In response to reviewers portion of the document, the authors indicate: “… based on their gender and racial composition, the NetHealth project team randomly selected 730 students …” It is not clear how this selection took place and what steps were taken to prevent sampling bias. But more importantly I do not understand why this information is not included in the body of the manuscript. What were reasons/criteria chosen to select that 730 out of 2007 freshmen*

*enrolled that year?*

––––––––––––––––––––––––––––––––––––––––––––––––––––

This is a great question. The NetHealth project was supported by the National Institute of Health (NIH). The project team made an estimation of the maximum number of Fitbit devices that could be distributed among the participants based on the NIH budget, and the answer was 730. We now include these details on pages 7-8 in the revised manuscript.

*2.2 Did they aim for a specific percent of let's say Asian students? Did they aim for a specific percent of male vs female?*

––––––––––––––––––––––––––––––––––––––––––––––––––––

Yes. The NetHealth project team adopted a stratified sampling strategy and aimed for a specific percentage of each gender-race strata based on the sampling frame. We now clarify this on page 8 in the revised manuscript.

*2.3 Please include this information in the body of the manuscript.*

––––––––––––––––––––––––––––––––––––––––––––––––––––

We now include the following information on pages 7 to 8 in the revised manuscript:

“In fall 2015 the University of Notre Dame admitted 2007 full-time freshmen, among which 1,069 (or 53%) were male students, 938 (or 47%) were female students, 1,352 (or 67%) were non-Hispanic white students, 219 (or 11%) were Hispanic students, 80 (or 4%) were non-Hispanic African-American students, 111 (or 6%) were non-Hispanic Asian-American students, 3 (or 0%) were non-Hispanic American Indian or Alaska Native students, 93 (or 5%) were non-Hispanic students with two or more races, 143 (or 7%) were students of other races, and 6 (or 0%) were students with race/ethnicity unknown (the detailed sampling frame is available from https://www3.nd.edu/~instres/CDS/2015-2016/CDS_2015-2016.pdf.) The NetHealth project team adopted a stratified sampling strategy and aimed for a specific percentage of each gender-race strata based on the sampling frame. The project team also made an estimation of the maximum number of Fitbit devices that could be distributed among the participants based on the NIH budget and sent out invitations to 730 students. Finally, 692 students accepted the invitations and completed the assent forms.”

*3. In response to reviewers portion of the document, the authors state that there was no exclusion criteria but at the same time they indicate that students identifying themselves to be in poor health or having disabilities were excluded from the model. These exclusion criteria should be included in the body of the manuscript.*

––––––––––––––––––––––––––––––––––––––––––––––––––––

We are sorry for the confusion resulting from our previous response to reviewers. The 10 (or 1.45%) students self-reporting poor health status and 13 (or 1.88%) students with physical disabilities were not excluded from our model. We meant to say we did not include “Health status” and “Physical disability” as explanatory variables in our model.

*4. It would be appropriate to include in the body of the manuscript the age range of the subjects or their average age and its standard deviation.*

––––––––––––––––––––––––––––––––––––––––––––––––––––

The ages of 99.6% participants ranged from 17 to 19 when they joined the project in 2015, with a mean of 18.4 and standard deviation of 1.8. We now report these numbers on page 10 in the revised manuscript.

*5. Regarding considering international student as a classification under racial background/ethnicity, I do understand the limitation the authors had to work by; regardless, please find some other form of classification that could apply.*

––––––––––––––––––––––––––––––––––––––––––––––––––––

We now classify “International student” as “Other races” in the revised manuscript.

**Reviewer 2**

*In my question about novelty and how this study can be differentiated from previous knowledge on the topic, the authors responded by citing a 2005 review paper. It sounds like an old reference to base the novelty of a 2020 contribution.*

––––––––––––––––––––––––––––––––––––––––––––––––––––

We thank the reviewer for this suggestion. We now add Barnett et al. (2014), Sallis et al. (2015), Yen & Li (2019), Scott (2020), and Rowlands (2020) to address the most previous knowledge on the topic as well as gaps in literature.

**References**

Barnett NP, Ott MQ, Rogers ML, Loxley M, Linkletter C, Clark MA. Peer associations for substance use and exercise in a college student social network. Health Psychol. 2014; 33: 1134–1142.

Rowlands AV. Measuring physical activity with body-worn accelerometers. In: Brusseau TA, Fairclough SJ, Lubans DR, editors. The Routledge Handbook of Youth Physical Activity. New York, NY: Routledge; 2020. pp. 330–346.

Sallis JF, Owen N, Fisher E. Ecological models of health behavior. In: Glanz K, Rimer BK, Viswanath K, editors. Health Behavior: Theory, Research, and Practice, 4th Edition. San Francisco, CA: Jossey-Bass; 2015. pp. 465–486.

Scott JJ. Pedometers for measuring physical activity in children and adolescents. In: Brusseau TA, Fairclough SJ, Lubans DR, editors. The Routledge Handbook of Youth Physical Activity. New York, NY: Routledge; 2020. pp. 315–329.

Yen HY, Li C. Determinants of physical activity: A path model based on an ecological model of active living. PLoS ONE. 2019; 14: e0220314.
